# Parental Depression and Leisure Activity Engagement on Children’s Gaming Disorder: A Dyadic Study

**DOI:** 10.3390/ijerph19105880

**Published:** 2022-05-12

**Authors:** Yee-Tik Lam, Cecilia Cheng

**Affiliations:** Department of Psychology, The University of Hong Kong, Hong Kong, China; edithlamlyt@connect.hku.hk

**Keywords:** excessive gaming, gaming addiction, depression, mental health, parent-based programs, parent-child dyad

## Abstract

Nowadays, playing both online and offline video games is a popular leisure activity among youngsters, but excessive gaming activity engagement may lead to gaming disorder that disrupts daily functioning. Identifying risk and protective factors of this emerging problem is thus essential for devising prevention and intervention strategies. This mixed-method, cross-sectional study aimed to examine the roles of parental depressive symptoms and children’s leisure activity engagement on children’s gaming disorder symptoms. Furthermore, the moderating roles of risky and protective leisure activity engagement were investigated. The sample comprised 104 parent-child dyads recruited from a population-based survey (parents: *M*_age_ = 45.59 years, *SD* = 6.70; children: *M*_age_ = 11.26 years; *SD* = 4.12). As predicted, parental depressive symptoms and children’s gaming activity engagement were positively associated with children’s gaming disorder symptoms, whereas children’s literacy activity engagement was negatively associated with these symptoms. Moreover, engagement in these two types of leisure activity moderated the association between parental depressive symptoms and children’s gaming disorder symptoms in distinct manners, further indicating literacy activities as beneficial and gaming activities as risk-enhancing. These new findings imply that parental depressive symptoms and children’s leisure activity engagement should be considered when designing parent-based programs for gaming disorder prevention and intervention.

## 1. Introduction

Gaming is one of the most popular leisure activities among children and adolescents. In a recent large-scale survey conducted in Hong Kong, more than 80% of the children and adolescent respondents reported playing both online and offline video games in their spare time [1]. Previous research has documented certain psychological and physical benefits of engaging in gaming activities, such as enhancing attention and working memory, expanding gamers’ social network and social capital, and improving physical health [2,3,4].

Despite the benefits of gaming, compulsive engagement in gaming activities can increase vulnerability to a myriad of mental health issues, such as emotion dysregulation, depression, anxiety, and hostility [5,6,7,8]. In addition, the excessive amount of time devoted to gaming activities can also influence psychological functioning in various life domains, such as family relations, academic studies, social life, and physical health [9,10,11]. In view of these adverse consequences, it is imperative to identify risk and protective factors of excessive gaming activity engagement to enrich our understanding of the etiology, such that preventive strategies can be devised for effective interventions.

### 1.1. Psychopathology of Excessive Gaming Activity Engagement

In view of the adverse consequences, internet gaming disorder was first included in the fifth revision of the Diagnostic and Statistical Manual of Mental Disorders (DSM-5) as a condition recommended for further research [12]. More recently, World Health Organization has officially recognized it as a mental health problem, and named it as gaming disorder (GD) in the eleventh revision of the International Classification of Diseases (ICD-11) [13]. According to the diagnostic criteria stated in the ICD-11, GD is categorized as an addictive behavior with a persistent or recurrent pattern of online or offline gaming behavior. More importantly, three diagnostic criteria also need to be met, including (a) having impairment in regulating gaming behavior, (b) prioritizing gaming to the extent that overrides activities in other life domains, and (c) failure in putting a halt to gaming even if undesirable consequences occur, with significant impairment in certain life domains [14].

A myriad of studies have been conducted to examine the prevalence, mechanisms, and outcomes of GD, but most studies have recruited adult samples [15]. A smaller body of studies have been conducted among non-adults, yet most have focused on adolescents [7,11]. This is probably because the DSM-5 states that the onset of GD is in adolescence [12]. However, a study showed that early exposure of online games among South Korean primary school students increases the likelihood of being diagnosed with GD in adolescence [16], suggesting the importance to study the emerging problem of GD in early childhood. The expansion of research to younger samples can advance knowledge for designing programs for early prevention and intervention of GD. To fill this knowledge gap, the present study is the first to examine the prevalence of GD across the entire span of childhood and adolescence (i.e., age ranges from 3 to 17) for facilitating comparisons among preschoolers, school-aged children, and adolescents.

### 1.2. Parental Depressive Symptoms and Gaming Disorder in Children

According to a systematic review [11], the development of GD is attributable to both internal factors (e.g., biological and psychological vulnerabilities) and external factors (e.g., family income, interpersonal deficits). Similar conclusions are also derived from another more recent review, which identifies some internal (e.g., being a male, having deficits in attention and emotional regulation) and external (e.g., having poor family relations) risk factors [17].

Both of these reviews have similarly shown the family environment as a major external risk factor of GD. When studying familial factors associated with GD, most researchers have examined family functioning and dynamics, such as parenting style and parental role models [18,19,20]. However, scant research attention has been paid on examining parents’ psychological problems as a potential risk factor of their children’s GD. As shown in a systematic review of studies on familial factors of GD in adolescents [21], only one study has examined parental mental health status [22].

A major aim of this study is to investigate parents’ depressive symptoms and its association with their children’s GD symptoms. We predicted a positive association parents’ depressive symptoms and children’s GD symptoms, since previous studies have revealed the influential role of parental psychological well-being on children’s mental health and problematic behaviors. Specifically, parental psychopathology significantly predicts both internalizing and externalizing problems in their children [23,24,25]. In this light, parental depression is proposed as a risk factor of GD. Previous studies have shown that this factor has led to many adverse outcomes in youths across different developmental stages [26,27]. For preschoolers living with parents diagnosed with depression, parental depressive symptoms have been found to have detrimental effects on children’s emotion regulation, behavioral inhibition, cognitive functioning, and parent-child attachment [28]. For children and adolescents, parental depressive symptoms have been found to predict poor academic performance, interpersonal issues, poor physical conditions, and internalizing and externalizing problems [26]. Taken together, it is reasonable to infer a positive association between parents’ depressive symptoms and their children’s GD symptoms.

### 1.3. Leisure Activity Engagement and Gaming Disorder among Children

While parents’ mental health status may play a distal role in their children’s GD, children’s activity engagement during spare time may be more directly related to GD symptoms. Another major aim of this study is to examine children’s engagement in leisure activities and its association with their GD symptoms. Surveys have documented that 91% of U.S. youngsters aged between 2 and 17 have engaged in gaming activities [29], and 80% of Hong Kong children and adolescents are regular gamers [1]. When individuals spend an excessive amount of time in gaming activities, their susceptibility to GD increases [30,31,32]. According to the time displacement theory [33], the total amount of time spent on daily activities is constant, implying that people spending time in a particular activity will decrease their engagement in other activities. In line with this theory, findings have documented that individuals with greater engagement in gaming activities tend to experience an increase in depressive symptoms over time due to a reduction in social interactions and family support [34].

In light of the time displacement theory and its related empirical evidence, an analysis of gamers’ engagement in leisure activities may be a promising approach that advances the understanding of GD. Studies have shown that individuals at a higher risk of developing GD or those diagnosed with GD are characterized by a narrower span of leisure activities and different preferences for the types of leisure activities than those without such GD problems [35,36]. These studies investigated the consequences of lifestyle owing to GD [37,38], but the lifestyle in terms of leisure activities may also be antecedents rather than consequences. For example, individuals at higher (vs. lower) risks for GD tend to have a greater preference in other screen time activities, especially those related to gaming, in their spare time [36]. Hence, engagement in gaming activities, other screen time activities, or both are proposed to be positively associated with GD symptoms.

A different line of studies also provided support for the time displacement theory by revealing that engagement in activities other than gaming and other screen time activities tends to reduce susceptibility to GD. In a longitudinal study of a large sample of emerging adults [39], a negative association is obtained between engagement in sport activities and GD symptoms. Similar findings are obtained in some studies that focused on internet addiction, an umbrella term that comprises an array of information technology addiction including GD [40]. Specifically, internet addiction symptoms are found to be negatively associated with engagement in sport activities, as well as family and outdoor activities [41,42]. In addition, a recent study has shown that having a reading habit, a literacy leisure activity, is associated with a lower risk of developing internet addiction [43].

Taking these theoretical postulations and empirical findings into account, we propose that susceptibility to GD may vary by the type of leisure activity engaged in. Specifically, engagement in some leisure activities—gaming and other screen time activities—is risk-enhancing, that is, positively associated with GD symptoms, whereas engagement in other leisure activities—literacy and sport activities—is protective, that is, negatively associated with GD symptoms.

### 1.4. Interplay of Parental Depressive Symptoms and Children’s Leisure Activity Engagement

As mentioned in the previous sections, the present study puts forward that both parental depressive symptoms and children’s leisure activity engagement may play a role in children’s GD symptoms. Although parental depressive symptoms may be positively associated with children’s GD symptoms, the magnitude of this association may vary by the type of leisure activities children engaged in. Specifically, we propose that engagement in gaming or other screen time activities may magnify this hypothesized positive association, whereas engagement in literacy or sport activities may buffer such an association.

Engagement in literacy and sport activities may be related to lower risks of GD, since such engagement tends to bolster self-efficacy and psychological wellbeing [44,45,46]. For instance, some studies have revealed that sport activity engagement can improve positive emotional experiences, quality of interpersonal relations, and a sense of social connectedness [47,48]. Studies also indicate that engagement in some literacy activities (i.e., reading, art, and music) can protect individuals from developing internalizing and externalizing problems [44,49]. All of these desirable psychosocial changes are found to lower risks of psychological distress for participants characterized by biological vulnerabilities or a low socioeconomic status. As leisure activity engagement is an understudied topic in GD research, this study thus investigates the hypothesized moderation effects of children’s leisure activity engagement in the positive association between parental depressive symptoms and children’s GD symptoms.

### 1.5. Overview of the Present Study

The present study adopted a dyadic approach to test the hypothesized associations among parental depressive symptoms, children’s leisure activity engagement, and children’s GD symptoms. To broaden the generalizability of the present findings across different age groups, the children sample comprises preschoolers, school-aged children, and adolescents. In this study, we first investigated the prevalence of GD among these three age groups. Then, we will test the hypothesized roles of parental depressive symptoms and children’s leisure activity engagement as well as the interactions of these factors on children’s GD symptoms. Specifically, the following hypotheses were tested:

**Hypothesis** **1.** *Parental depressive symptoms will be positively associated with children’s GD symptoms.*

**Hypothesis** **2.** *Children’s engagement in two clusters of risky leisure activities (i.e., gaming and other screen time) will be positively associated with children’s GD symptoms.*

**Hypothesis** **3.** *Children’s engagement in two clusters of protective leisure activities (i.e., literacy and sport) will be negatively associated with children’s GD symptoms.*

**Hypothesis** **4.** *Children’s engagement in two clusters of risky leisure activities (i.e., gaming and other screen time) will strengthen the positive association between parental depressive symptoms and children’s GD symptoms.*

**Hypothesis** **5.** *Children’s engagement in two clusters of protective leisure activities (i.e., literacy and sport) will weaken the positive association between parental depressive symptoms and children’s GD symptoms.*

## 2. Materials and Methods

### 2.1. Research Design and Participants

The present study adopted a mixed-method, cross-sectional design. The data were derived from a population-based survey, namely the Hong Kong Mental Health and Digital Wellness Survey. In this survey, a computer-assisted telephone interview was conducted on a randomly drawn community sample of 1006 Hong Kong Chinese participants (384 males and 622 females; *M*_age_ = 54.62, *SD* = 18.17, range: 18–94). As the present study adopted a dyadic approach, only the data of parent-child dyads were retrieved from the dataset. To obtain the dyadic data, we adopted the following a priori inclusion criteria: (a) parents with children who were between 3 and 17 years old, (b) no previous history of psychiatric disorder for both parents and their children, and (c) willingness to give consent for both parents and their children before the survey began.

On the basis of these inclusion criteria, the data of 105 parent-child dyads were extracted. However, the data of one dyad were removed because there were no data concerning children’s GD symptoms. The final sample contained 104 parent-child dyads. The parent group consisted of 43 males and 61 females with an average age of 45.59 years (*SD* = 6.69), whereas the child group consisted of 51 males and 53 females with an average age of 11.26 years (*SD* = 4.12). The parent group did not significantly differ from the entire group of survey participants regarding the gender ratio [χ^2^(1) = 0.50, *p* = 0.48] and level of depressive symptoms [*U* = 44,445.50, *z* = −0.07, *p* = 0.95]. The parent group was younger than the entire group of survey participants, mainly because the latter included elderly participants aged between 61 and 94 as well. However, when making comparisons between the parent group and the survey sample with adult participants only (i.e., aged between 18 and 60), there were no age differences between the two adult groups, *U* = 22,641.00, *z* = −1.02, *p* = 0.31.

### 2.2. Measures of Parent- and Children-Report

The study variables were assessed by both parent- and children-ratings. Specifically, parents were instructed to report their children’s leisure activity engagement through an open-ended question. In addition, parents reported their own depressive symptoms measured by the short form of the Center of Epidemiologic Studies Depression Scale (CESD-10), whereas their children reported their own GD symptoms measured by Gaming Disorder Test (GDT). 

The present study adopted a mixed-method design that utilized both quantitative and qualitative methods. Quantitative measures were used to assess parental depressive symptoms, children’s GD symptoms, and demographic information, whereas children’s leisure activity engagement was assessed by the qualitative method. For statistical analysis, the qualitative data were coded and transformed to quantitative data after the data collection. Details of all these measures and the coding process were described below.

#### 2.2.1. Parental Depressive Symptoms

Parent participants rated their own levels of depressive symptoms through the Chinese version of the short form of the CESD-10 [50,51]. This instrument was made up of 10 items to measure depressive symptoms, including three items on depressive affect, two items on positive affect, and five items on somatic symptoms related to depression. Each item was rated on a 4-point Likert scale, with 0 indicating “*rarely or none of the time*” and 3 indicating “*all the time*”. The scores of those items assessing positive affect were reversed scored. The composite score of CESD-10 ranged from 0 to 30, with a higher score indicating a greater severity of depressive symptoms. The Chinese CESE-10 was found to display good reliability and validity [50]. The Cronbach’s alpha of this scale in this study is 0.63.

#### 2.2.2. Children’s Leisure Activity Engagement

Parent participants also reported their children’s engagement in leisure activities through an open-ended question that asked the respondents to list three leisure activities their children engaged in during free time. The open-ended responses were coded according to a combination of concept- and data-driven strategies [52]. Specifically, a trained coder first reviewed the entire list to become familiarized with the participants’ answers at the pilot stage. The coder created the codes and categories based on the nature of activities through a data-driven strategy, and then discussed with other research team members who were not involved in the coding process. As a result, four categories were created based on the literature review and the data pattern, namely gaming, other screen time, literacy, and sport activities (see Table 1). All of these responses were dummy coded, with “1” indicating that the participants’ children had engaged in a particular type of leisure activity, and “0” indicating that their children did not engage in it. This dichotomous variable was labeled as high-level activity engagement and low-level activity engagement respectively, based on the consideration that just because a certain type of leisure activity was not mentioned in the free response did not necessary mean that the participants did not engage in that leisure activity at all, but rather the tendency to engage less in that activity during the report period. 

In order to evaluate the temporal reliability of coding, the responses were coded twice within a month [53,54]. To resolve the discrepancies in the two sets of coding, the coder discussed with the corresponding author in a post-hoc meeting to locate the best-fit category. 

#### 2.2.3. Children’s Gaming Disorder Symptoms

Children’s ratings of their own GD symptoms were measured using the Chinese version of the GDT [55]. This measure comprised four items to assess the levels of gaming disorder symptoms based on the ICD-11 framework [14,55]. All of these items were rated on a 5-point Likert scale, ranging from 1 (“never”) to 5 (“very often”). The composite score ranged from 4 to 20, with a higher score indicating a greater symptom severity and a higher risk of developing gaming disorder [55]. The psychometric properties of the Chinese GDT were demonstrated in previous research [14,55]. 

It is important to note that GDT is not a diagnostic tool of GD, and the main purpose of using this instrument is to assess the severity of GD and the accompanying detrimental effects to the gamer’s daily life. However, for research purposes to investigate the prevalence of GD in the children sample, we followed the scale developers’ recommendations by adopting the strict polythetic classification scheme, with a coding of a 4 (“*often*”) or 5 (“*very often*”) for any of the four items being categorized as the endorsement of the corresponding GD criterion. Adopting this (≥ 4 or 5) classification scheme allowed researchers to distinguish between risky and non-risky gamers [55]. The Cronbach’s alpha of the GDT in this study is 0.91.

#### 2.2.4. Demographic Variables

At the end of the survey, the respondents provided answers to a set of questions for yielding the following demographic data: parent’s age, gender, and education level, as well as their children’s age and gender. In the stage of data coding, child participants were further categorized into three age groups: preschoolers (age 3–5), school-aged children (age 6–12), and adolescents (age 13–17).

### 2.3. Statistical Analysis

The data were analyzed by IBM SPSS Statistics for Windows, Version 27.0 (IBM Corp, Armonk, NY, USA). Prior to the performance of statistical analysis, the normality assumption was checked using skewness, kurtosis, and a Shapiro–Wilk test. Histograms, box plots, and Q-Q plots were also generated to allow for an observation of the variables’ score distribution. 

Preliminary analyses were conducted to explore the potential demographic differences in the study variables. To analyze those variables whose scores were not normally distributed, the following non-parametric tests were conducted: chi-square test of independence, Mann–Whitney test, and Kruskal–Wallis tests. To analyze those variables with an approximately normal distribution of scores, the parametric tests of independent-samples *t*-test and Pearson correlation were conducted. 

Before the hypothesis testing using regression analysis, regression assumption testing was also conducted. For testing the first three hypotheses, a hierarchical multiple regression analysis was performed to test the hypothesized associations between the predictor variables (demographic variables, parental depressive symptoms, and children’s engagement in various leisure activities) and the criterion variable (children’s GD symptoms). The scores of all continuous variables were centered before entering into a regression model [56]. For testing Hypotheses 4 and 5, Model 1 of the PROCESS macro for Windows Version 4.0 [57] was performed to examine the hypothesized moderating effect of children’s leisure activity engagement on the association between parental depressive symptoms and children’s GD symptoms. As recommended by Hayes [56], the confidence intervals (CIs) were computed based on 5000 bootstrap samples to test the validity of the effects. A *p* level of 0.05 was used to determine statistical significance for all of these analyses.

## 3. Results

### 3.1. Preliminary Analysis

The normality test results showed that children’s age, as well as the scores of parental depressive symptoms and children’s GD symptoms, were not normally distributed [*W*’s(104) < 0.21, *p*’s < 0.001]. The observation of histograms, box plots and Q-Q plots also showed the same pattern of results. The descriptive statistics of the demographic and study variables are presented in Table 2 and Table 3, respectively.

For detecting differences in parent’s age, the independent-samples *t*-tests showed that children whose parents were older had greater engagement in gaming activities than those whose parents were younger, *t* (102) = −3.43, *p* = 0.001, Cohen’s *d* = −0.66.

For examining differences in parent’s education level, the chi-square test revealed significant differences in children’s literacy activity engagement, *χ*^2^(1, *n* = 104) = 3.86, *p* = 0.05, φ = 0.19. Specifically, children whose parents were degree-holders were more likely to engage in literacy activities than those whose parents were non-degree holders.

For investigating differences in children’s age, the chi-square test revealed significant differences in children’s gaming activity engagement, χ^2^(2, *n* = 104) = 7.00, *p* = 0.03, Cramer’s *V* = 0.26. Specifically, adolescents reported greater engagement in gaming activity than both school-aged children and preschoolers.

For studying differences in children’s gender, the Mann–Whitney test showed that male children reported significantly higher levels of GD symptoms than their female counterparts, *U* = 977.50, *z* = −2.52, *p* = 0.01, *r* = −0.25. The chi-square test further revealed significant gender difference in literacy activity engagement, χ^2^(2, *n* = 104) = 6.01, *p* = 0.01, φ = 0.24. Female children were more likely to engage in literacy activities than their male counterparts. In contrast, an opposite pattern of gender difference was found for sport activity engagement, χ^2^(2, *n* = 104) = 6.55, *p* = 0.01, φ = −0.25. Male children were more likely to engage in sport activities than female children.

The Pearson correlation analysis showed a negative association between children’s literacy activity engagement and their gaming activity engagement (*r* = −0.27, *p* = 0.005)*,* as well as a negative association between children’s literacy activity engagement and their sport activity engagement (*r* = −0.26, *p* = 0.007). These results indicate that children with greater engagement in literacy activities were less likely to engage in gaming and sport activities. These results provide further support for the postulations of the time displacement theory.

### 3.2. Risk of Gaming Disorder among Age Groups

The proportion of children at risk for GD was significantly different among the three age groups. Specifically, the school-aged children group had the highest proportion of children at risk for GD (32%), which was around three times higher than those of preschoolers (9%) and adolescents (11%).

### 3.3. Hierarchical Regression Analysis

The multiple assumptions of regression were checked before testing the hypotheses. Specifically, the linear associations of the independent and dependent variables were examined, and this assumption was met. As for the analysis of collinearity statistics, the VIF scores were all well below 7, with the tolerance score above at least 0.51 in all regression models. Furthermore, the obtained value on the Durbin–Watson statistics were close to 2. The plots of standardized residuals versus standardized predicted values showed no obvious funneling, indicating that the assumption of homoscedasticity was met. Moreover, the P-P plots for the regression model showed a normality of the residuals. Cook’s Distance values were all under 1, indicating that individual cases were not unduly influencing the regression model.

A hierarchical regression analysis was performed to investigate factors that were associated with children’s GD symptoms, without controlling for the demographic variables. The results are summarized in Table 4. Referring to this table, the variable of parental depressive symptoms was entered into the first step, and this variable accounted for 5% of the variance in children’s GD symptoms (*p* = 0.03). Then, engagement in all four types of leisure activity were entered into the second step, and this set of variables explained 25% of the variance above and beyond the variable of parental depressive symptoms. Overall, this model accounted for 30% of the variance in children’s GD symptoms (*p* < 0.001).

Another hierarchical regression analysis was conducted to investigate factors that were associated with children’s GD symptoms, but this regression model differed from the previous one by controlling for the effects of demographic variables. The results are summarized in Table 5. Referring to this table, all five demographic variables were entered into the first step, and this set of demographic variables explained 11% of the variance in children’s GD symptoms (*p* = 0.04). Then, the variable of parental depressive symptoms was entered into the next step, and this variable accounted for 7% of the variance above and beyond that accounted for by the demographic variables (*p* = 0.007). Children’s engagement in all four types of leisure activities were entered into the final step, and this set of variables explained a further 19% of additional variance (*p* < 0.001). Overall, the three clusters of variables explained 36% of the variance in children’s GD symptoms (*p* < 0.001).

In summary, these results were consistent with Hypothesis 1 in indicating that the higher the levels of depressive symptoms reported by parents, the higher the levels of GD symptoms for their children, and vice versa. For the two hypothesized risky leisure activities, only engagement in gaming activities, but not other screen time activities, was positively associated with children’s GD symptoms. Similarly, for the two hypothesized protective leisure activities, only engagement in literacy activities but not sport activities was negatively associated with children’s GD symptoms. Taken together, the findings provided partial support for both Hypotheses 2 and 3 in indicating that children with higher levels of GD symptoms tended to engage in more gaming activities but less literacy activities.

### 3.4. Moderation Analysis

The hypothesized moderating effects of children’s leisure activity engagement were then tested. Among the four types of leisure activities, only two of the moderation effects were significant. First, children’s gaming activity engagement positively moderated the hypothesized positive association between parental depressive symptoms and children’s GD symptoms, *B* = 0.40, *t* = 2.23, *p* = 0.03.

**Figure 1:**
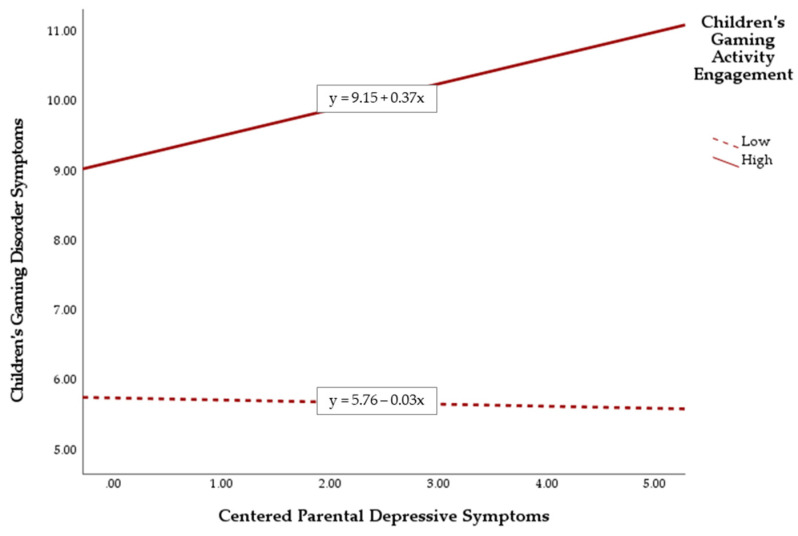
Plot of simple slope analysis for unpacking the significant interaction between parental depressive symptoms and children’s gaming activity engagement.

The results of simple slope analysis are depicted in Figure 1. Referring to this figure, the positive association between parental depressive symptoms and children’s GD symptoms was stronger among children with greater engagement in gaming activities during leisure time than those with less such engagement. A post-hoc regression analysis was performed to further unpack this moderation effect, and the results revealed that the positive association between parental depressive symptoms and children’s GD symptoms was significant only for children who engaged in gaming activities [*B* = 0.37, *t* = 2.61, *p* = 0.01)], but not those without such engagement [*B* = −0.03, *t* = −0.27, *p* = 0.79)].

Second, children’s literacy activity engagement negatively moderated the hypothesized positive association between parental depressive symptoms and children’s GD symptoms, *B* = −0.48, *t* = −2.53, *p* = 0.01.

**Figure 2:**
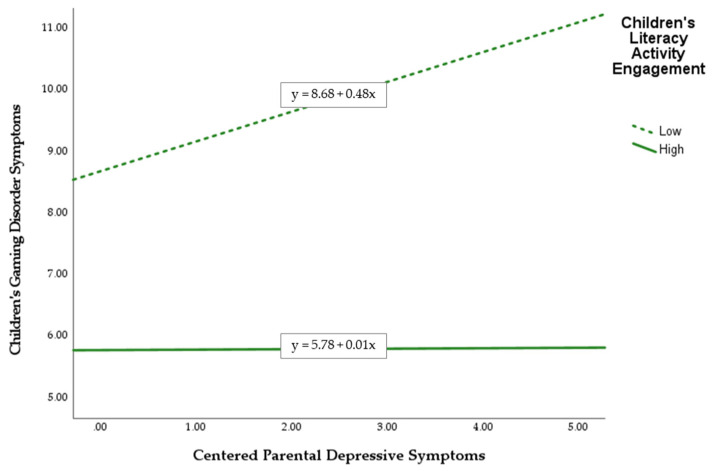
Plot of simple slope analysis for unpacking the significant interaction between parental depressive symptoms and children’s literacy activity engagement.

The results of simple slope analysis are shown in Figure 2. This figure indicates that the positive association between parental depressive symptoms and children’s GD symptoms was weaker for children with greater engagement in literacy activities during leisure time, compared with their counterparts with less engagement in this type of activity. The post-hoc test further indicated that the positive association between parental depressive symptoms and children’s GD symptoms was significant only for children who did not engage in literacy activities (*B* = 0.49, *t* = 3.53, *p* = 0.001), but not for those who engage in literacy activities (*B* = 0.01, *t* = 0.07, *p* = 0.95).

In summary, the present results provided partial support for Hypotheses 4 and 5 in revealing that only children’s gaming activity engagement and their literacy activity engagement were significant moderators of the positive association between parental depressive symptoms and children’s GD symptoms. Despite the partial support, the results were largely in line with our predictions in revealing the former as a risky type of leisure activity that magnified this association, whereas the latter was a protective type that buffered the association.

## 4. Discussion

### 4.1. Principal Findings

The present study adopted a dyadic approach in investigating the emerging problem of GD across the span of childhood and adolescence. Among the three age groups, school-aged children tend to be threefold more at risk for GD than preschoolers and adolescents. In addition, this study aimed to investigate the associations among parental depressive symptoms, children’s leisure activity engagement, and children’s GD symptoms. As predicted, the findings indicate a positive association between parental depressive symptoms and children’s GD symptoms, both with and without controlling the demographic variables. More importantly, the positive association between parental depressive symptoms and children’s GD symptoms tends to be stronger for children with greater (vs. less) engagement in gaming activities, providing support for the risk-enhancing role of gaming activity engagement. On the contrary, the positive association between parental depressive symptoms and children’s GD symptoms tends to be weaker for children with greater (vs. less) engagement in literacy activities, providing support for the protective role of literacy activity engagement.

#### 4.1.1. Age Differences in GD Risk and Gaming Activity Engagement

Extending the literature by examining the problem of GD in younger samples, our findings indicate that children as young as preschoolers may also be susceptible to GD. Although the DSM-5 states that GD is the most prevalent in adolescents with reference to other addictive behaviors [12]. we propose that this inconsistency may be due to the scant research effort that examines the problem of GD in young populations of preschoolers and school-aged children [7,11]. More research attention should be devoted to investigating this emerging problem across the entire span of childhood and adolescence.

It is noteworthy that the rate of high-risk GD in the school-aged children group tends to be higher than those obtained in some of the studies, which indicate that the global prevalence of GD is around 2% to 5% [58,59]. However, some systematic reviews estimate higher prevalence of GD that may reach up to 28% to 58% [60,61]. Such discrepancies in the estimated prevalence may be attributable to the differences in the classification criterion of high-risk GD across different assessment tools [14]. Researchers should conduct more large-scale population-based surveys on representative, demographic diverse samples to compare the prevalence yielded by an array of existing tools of GD assessment.

Although school-aged children are found to be the most at risk among the three groups of participants, it is interesting to note that adolescents tend to have the highest frequency of engagement in gaming activities. As self-regulation plays a pivotal role in the development of GD [62], adolescents may be less susceptible to GD, despite a higher engagement in gaming activities due to their greater ability to self-regulate than those of their younger counterparts. This notion is consistent with existing evidence that reveals the more developed executive functioning among adolescents compared with their younger counterparts [63]. In this light, adolescents may have better self-control over their gaming behavior and thus be less vulnerable to GD compared with school-aged children. Another possibility is that adolescents may be more occupied with other commitments or have greater mobility to engage in other leisure activities, especially those that take place outside home [64,65,66]. As adolescents are more apt to multitasking [67], they may engage in an array of tasks or chores while gaming, and thus may focus less on gaming activities compared with their younger counterparts who have less commitments. Their relatively less attention may lower their susceptibility to GD than school-age children.

#### 4.1.2. Leisure Activity Engagement and Gaming Disorder

This study is among the few to study the role of leisure activity engagement on GD symptoms, and the findings indicate that children’s gaming activity engagement is positively associated with their GD symptoms. This finding is in line with the predictions of the time displacement theory [33], proving our prediction of gaming activity engagement as a risk factor of GD.

More importantly, our findings further indicate a negative association between children’s literacy activity engagement and GD symptoms. In addition, such engagement moderates the positive association between parental depressive symptoms and children’s GD symptoms. This new finding indicates that the positive association between these two types of symptoms is weaker for children with greater engagement in literacy activities in their spare time. In the literature, literacy activity engagement has been shown to improve emotion regulation, facilitate positive emotions, bolster self-efficacy, and mitigate psychological distress [68,69,70]. All of these desirable changes brought by literacy activity engagement may serve as a protective factor from developing internalizing or externalizing problems [44,46], including GD that is the target problem examined in the present study.

The findings are inconsistent with our predictions in revealing non-significant associations of children’s GD symptoms with both sport activity engagement and other screen time activity engagement. The discrepancies in the pattern of findings regarding gaming versus other screen time activity engagement suggest that future research should distinguish these two types of leisure activity, which have often been conceptualized as a single study variable in the literature [71,72,73]. As shown in our study, engagement in other screen time activities, such as watching movies or television, is unrelated to GD symptoms. These findings imply the importance of adopting a nuanced approach when investigating leisure activity engagement. In this approach, gaming and other screen time activities should be tested separately; and in the same vein, it is possible to further break down sport activity engagement into more refined constructs such as solitary and group sport activities. The adoption of a nuanced approach may further advance the understanding of the understudied association between leisure activity engagement and the development of GD symptoms.

### 4.2. Demographic Characteristics and Gaming Disorder

Apart from the principal findings, the present findings also reveal that boys tend to report more GD symptoms than girls, a finding that is consistent with those of previous studies in revealing that boys are at higher risks of GD than girls [74,75]. In addition, the results indicate that children with older parents are more likely to engage in gaming activities. Such a finding may be due to a lack of knowledge in technology, gaming, or both among older parents, making it more difficult for them to monitor their children’s gaming behavior [19,76]. A survey documents that older (vs. younger) parents aged 50 years or above tend to have greater difficulties in parenting; and such age cohort differences are attributable to the rapid technological advancement, with older (vs. younger) parents having fewer concerns over excessive smartphone use in their children [76].

This study also shows that children with parents who are degree holders (vs. non-degree holders) are more likely to engage in literacy activities. As parental education level is a core indicator of socioeconomic status [77,78], this finding implies that families with a lower socioeconomic status are more likely to encounter the problem of GD. These underprivileged families thus need greater resources and support to tackle this emerging problem. Moreover, girls (vs. boys) are found more likely to engage in literacy activities, which are negatively associated with gaming activity engagement. These two pieces of findings may explain in part why boys tend to report higher levels of GD symptoms than girls, as literacy activity engagement is negatively associate with GD symptoms. Differentiating among the type of leisure activity is valuable in enriching our understanding of risk and resource factors of GD.

### 4.3. Practical Implications for Gaming Disorder Prevention and Intervention

The present new findings may have practical implications for mental health professionals, school personnel, and parents. In light of the prevalence of GD among preschoolers, we advocate that the prevention and intervention for GD should be implemented as early as possible. Specifically, literacy activities should be promoted in kindergarten to lower the risk of GD, especially the peak of GD risk is found to be the highest when children reach school-age in the present study. For fostering engagement in literacy activities, the school is a potential source to help promoting such GD-buffering activities through extracurricular activities or school programs [45,79,80].

A review of existing interventions for GD shows that many intervention programs adopt an individual approach with a sole focus on a single target group of clients, such as delivering cognitive behavior therapy for adolescents, as well as strengthening parenting and communication skills for parents [81,82]. However, the present findings show a robust positive association between parental depressive symptoms and their children’s GD symptoms, but there are no gender differences in the levels of depressive symptoms. In light of these findings, we propose that intervention strategies should be expanded to take care of the emotional needs of parents, in both genders. Previous work has only focused on maternal depressive symptoms, while paternal depressive symptoms have often been neglected [83,84,85].

Some interventions encourage parent involvement, yet most of them have focused mainly on parental monitoring rather than their psychological well-being [86,87,88]. For instance, the Game-Over Intervention, a parent-based-intervention on GD, emphasizes psychoeducation of GD and skill building related to parental care and monitoring [89]. This program is currently being conducted among parents of upper primary school students. We thus advocate the program beneficiaries to be expanded to include parents of lower primary school or kindergarten students. In addition, the program content can be extended to promote children’s literacy activities as well as strengthen parents’ self-care and social capital accrual.

The present study highlights that parents and children both encounter psychological problems, with parents afflicted by depressive symptoms while their children afflicted by GD symptoms. In this light, an intervention approach focuses on the family system, such as multidimensional family therapy, may be more beneficial to both parents and their children [89,90,91]. For example, an intervention program of PIPATIC (Programa Individualizado Psicoterapéutico para la Adicción a las Tecnologías de la Información y la Comunicación), specifically designed for adolescents with GD, consists of a module targeted on family communication, boundaries, and bonding [91]. This intervention has been shown promising effects on mitigating GD symptoms. Yet, as pointed out previously, the issue of parental mental wellness has not been addressed in the current program. Future design of parent- or family-based GD intervention programs should be incorporated components to take care of parental wellbeing into the protocol, with a reference to an existing Coping Power protocol designed for children with aggressive behavior [92].

### 4.4. Strengths, Limitations, and Future Research Directions

The present study has several strengths. First, the study examined the entire span of childhood and adolescence in the inquiry of GD, thus extending the literature that mostly focused on adolescents and adults [7,11]. Considerable differences have been identified among preschoolers, school-aged children, and adolescents. Second, the present dyadic study collects data using both parent- and children-reports in order to provide a more comprehensive perspective on psychological issues faced by different members in a family. A major advantage of including ratings by different parties is to minimize common method biases faced by the majority of studies, which rely largely on a single method of self-report in data collection [93]. Third, to the best of our knowledge, the present study is the first to investigate the moderation effect of children’s leisure activity engagement in the association between parental depressive symptoms and children’s GD symptoms. Leisure activity engagement is currently an understudied area in GD research, and more research effort in this area is encouraged. Fourth, we utilized a combination of data-driven and concept-driven strategies for analyzing children’s leisure activities. This method integrates empirical evidence with participants’ free responses, and such an integration enriches our understanding of the vast array of leisure activities children and adolescents engage in [52,94].

Some limitations of this study are also noteworthy, and some directions for future research are thus proposed. The study was conducted in Hong Kong and the sample comprises Chinese parent-child dyads. As there are considerable cultural differences in parenting style and experience of psychological well-being [95,96,97,98], the present findings may not be generalizable to parents and children residing in other cultural regions. Future studies should be conducted in different countries to evaluate the extent of generalizability in the present findings. Moreover, the present data were drawn from a population-based telephone survey that made it difficult to collect parent-child dyadic data. This is because the parents and their children should not only be present at the same time, but also consent to take part must be obtained from both parties. Similar to the problems faced by most other dyadic studies, we had very little control over the participant recruitment process, and the final sample size was somewhat smaller than our expectations. Moreover, this study focuses only on GD, and the findings may not necessarily be applicable to explain other types of information technology addiction, such as social media or smartphone addiction [99,100]. Finally, in this study, we did not explain the underlying mechanisms of how parental depressive symptoms and leisure activity engagement may contribute to the development of GD, and future studies may expand the scope of the present study by examining some psychological mechanisms to enhance the explanatory utility of findings.

## 5. Conclusions

In this article, we examined the phenomenon of GD and compared its symptom severity among different age groups of children. The main study aim focuses on investigating the associations between parental depressive symptoms and children’s GD symptoms, with engagement in various leisure activities as the moderators. The major findings indicate that the positive association between parental depressive symptoms and children’s GD symptoms tends to be stronger for children with greater (vs. less) engagement in gaming activities, but weaker for those with greater (vs. less) engagement in literacy activities.

In light of these new findings, we propose some potential explanations and call for an adaptation and expansion of the intervention design to mitigate children’s GD symptoms through both promoting children’s literacy activities, as well as taking care of parents’ emotional needs and bolstering their emotional well-being. Finally, we have proposed some directions for future research to increase the generalizability of findings by conducting studies in various cultural contexts and advance further understanding of the GD phenomenon by expanding the scope to the investigation of underlying psychological mechanisms.

## Figures and Tables

**Table 1:** Coding categories and children’s leisure activities reported by parent participants.

Coding Category	Children’s Leisure Activity
Gaming activities	playing video games
Other screen time activities	watching videos, watching television, chatting over the phone, watching YouTube, watching Netflix, watching movies, using iPad
Literacy activities	drawing, playing piano, reading, painting, listening to music, writing, going to the library
Sport activities	dancing, playing badminton, swimming, riding a bike, playing basketball, playing trampoline, tapping a ball, jogging, doing exercises, running, jumping, playing table tennis, cycling, playing kin-ball, playing ball games, soccer

**Table 2:** Demographic characteristics of parent and child groups.

Demographic Variable	*n* (*%*)	*M* (*SD*)	Range
Parent’s age	104	45.59 (6.69)	27–63
Parent’s gender			0–1
Female	61 (59%)		
Male	43 (41%)		
Parent’s education level			0–1
Non-degree holder	58 (56%)		
Degree-holder	46 (44%)		
Children’s age		11.26 (4.12)	3–17
Preschoolers	11 (11%)		
School-aged children	47 (45%)		
Adolescents	46 (44%)		
Children’s gender			0–1
Female	53 (51%)		
Male	51 (49%)		

**Table 3:** Descriptive statistics of study variables (*n* = 104).

Study Variable	*M* (*SD*)	Range
Parental depressive symptoms	7.14 (3.74)	0–18
Children’s gaming activity engagement	0.46 (0.50)	0–1
Children’s other screen time activity engagement	0.46 (0.50)	0–1
Children’s literacy activity engagement	0.39 (0.49)	0–1
Children’s sport activity engagement	0.40 (0.49)	0–1
Children’s GD symptoms	7.36 (3.90)	4–18

**Table 4:** Hierarchical regression model testing the role of parental depressive symptoms and leisure activity engagement on children’s GD symptoms (*n* = 104).

Predictor Variable	Step 1	Step 2
Parental depressive symptoms	0.22 *^1^	0.25 **
Children’s gaming activity engagement		2.79 ***
Children’s other screen time activity engagement		0.26
Children’s literacy activity engagement		−2.18 **
Children’s sport activity engagement		−0.23
*R* ^2^	0.05	0.30
*R*^2^ Change	0.05	0.25
*F* Change	4.82 *	8.75 ***

* *p* < 0.05, ** *p* < 0.01, *** *p* < 0.001.; ^1^ All values are unstandardized regression coefficients.

**Table 5:** Hierarchical regression model testing the role of demographic variables, parental depressive symptoms, and leisure activity engagement on children’s GD symptoms (*n* = 104).

Predictor Variable	Step 1	Step 2	Step 3
Parent’s gender ^2^	−0.93 ^1^	−1.14	−0.66
Parent’s age	0.09	0.08	0.04
Parent’s education level ^3^	−0.04	0.01	0.72
Children’s gender ^2^	−2.29 **	−2.53 **	−1.67 *
Children’s age	−0.04	−0.03	−0.13
Parental depressive symptoms		0.27 **	0.27 **
Children’s gaming activity engagement			2.80 ***
Children’s other screen time activity engagement			0.73
Children’s literacy activity engagement			−1.96 *
Children’s sport activity engagement			−0.13
*R* ^2^	0.11	0.18	0.36
*R*^2^ Change	0.11	0.07	0.19
*F* Change	2.44 *	7.64 **	6.75 ***

* *p* < 0.05, ** *p* < 0.01, *** *p* < 0.001.; ^1^ All values are unstandardized regression coefficients; ^2^ 0 = male, 1 = female; ^3^ 0 = non-degree holder, 1 = degree holder

## Data Availability

The data presented in this study are available on request from the corresponding author.
